# Dynamic DNA methylation reconfiguration during seed development and germination

**DOI:** 10.1186/s13059-017-1251-x

**Published:** 2017-09-15

**Authors:** Taiji Kawakatsu, Joseph R. Nery, Rosa Castanon, Joseph R. Ecker

**Affiliations:** 1 Plant Biology Laboratory, The Salk Institute for Biological Studies, La Jolla, CA 92037 USA; 2 Genomic Analysis Laboratory, The Salk Institute for Biological Studies, La Jolla, CA 92037 USA; 3 Institute of Agrobiological Sciences, National Agriculture and Food Research Organization, Tsukuba, Ibaraki 305-8602 Japan; 4 Howard Hughes Medical Institute, The Salk Institute for Biological Studies, La Jolla, CA 92037 USA

**Keywords:** *Arabidopsis thaliana*, DNA methylation, Embryogenesis, Germination, Dry seed

## Abstract

**Background:**

Unlike animals, plants can pause their life cycle as dormant seeds. In both plants and animals, DNA methylation is involved in the regulation of gene expression and genome integrity. In animals, reprogramming erases and re-establishes DNA methylation during development. However, knowledge of reprogramming or reconfiguration in plants has been limited to pollen and the central cell. To better understand epigenetic reconfiguration in the embryo, which forms the plant body, we compared time-series methylomes of dry and germinating seeds to publicly available seed development methylomes.

**Results:**

Time-series whole genome bisulfite sequencing reveals extensive gain of CHH methylation during seed development and drastic loss of CHH methylation during germination. These dynamic changes in methylation mainly occur within transposable elements. Active DNA methylation during seed development depends on both RNA-directed DNA methylation and heterochromatin formation pathways, whereas global demethylation during germination occurs in a passive manner. However, an active DNA demethylation pathway is initiated during late seed development.

**Conclusions:**

This study provides new insights into dynamic DNA methylation reprogramming events during seed development and germination and suggests possible mechanisms of regulation. The observed sequential methylation/demethylation cycle suggests an important role of DNA methylation in seed dormancy.

**Electronic supplementary material:**

The online version of this article (doi:10.1186/s13059-017-1251-x) contains supplementary material, which is available to authorized users.

## Backgrounds

DNA methylation is a DNA modification that can affect gene expression, transposable element (TE) activity, and heterochromatin formation. DNA methylation (mC) occurs in three distinct sequence contexts, CG and CHG (symmetric) and CHH (asymmetric); where H = C, A, or T. The reference plant *Arabidopsis thaliana* has four distinct DNA methylation pathways. Methylated CG (mCG) is maintained by DNA METHYLTRANSFERASE 1 (MET1) in a semi-conservative manner during DNA replication [1]. Methylated CHG (mCHG) is maintained by CHROMOMETHYLASE 3 (CMT3), which is targeted to DNA by recognizing H3K9 methylation [2, 3]. Methylated CHH (mCHH) is maintained by RNA-directed DNA methylation (RdDM). In RdDM, RNA polymerase IV (pol IV)-dependent 24-nucleotide (nt) or aberrant transcript dependent 21-nt small RNAs recruit DOMAINS REARRANGED METHYLTRANSFERASE 2 (DRM2) to target regions [4–6]. DRM2 catalyzes all contexts of DNA methylation. mCHG and mCHH are also maintained by CMT2 which recognizes H3K9 di-methylation and tri-methylation in deep heterochromatin [7, 8]. CMT2-dependent DNA methylation is associated with heterochromatin formation in Arabidopsis. In contrast, Arabidopsis has four closely related DNA demethylation enzymes: DEMETER (DME); REPRESSOR OF SILENCING 1 (ROS1) / DEMETER-LIKE 1 (DML1); DML2; and DML3 [9–12]. DME is required for genomic imprinting in the endosperm, whereas ROS1, DML2, and DML3 act in vegetative tissues. ROS1 antagonizes RdDM and RdDM-independent DNA methylation and may prevent spreading of DNA methylation from TEs to protein-coding genes [13]. ROS1 expression is positively regulated by proximal RdDM-dependent TE methylation [14, 15]. Therefore, active DNA methylation and demethylation are balanced in the cells.

Reprogramming is a phenomenon whereby chromatin modifications, such as DNA methylation and histone modifications, are erased and re-established during development. In mouse, two rounds of genome-wide mCG reprogramming occur during the life cycle [16]. Global demethylation occurs just after fertilization to erase the memory of the previous generation, except for genomic imprinting regions which are maintained. After subsequent global remethylation, the second round of global demethylation erases imprintings in the migrating primordial germline cells. In plants, DNA methylation reprogramming occurs in pollen [17, 18]. mCG and mCHG are retained, but mCHH is reduced in the microspore and sperm cells. In contrast, while mCG is reduced, mCHH is increased near the centromere in the vegetative cell. CG demethylation in the vegetative cell allows expression of TEs whose transcripts are then subject to processing into siRNAs [17, 19]. These epigenetically activated small RNAs move into sperm cells and reinforce mCHH for genomic imprinting and TE silencing [20]. After fertilization, MET1, CMT3, and RdDM pathways are highly active, promoting global hypermethylation in the torpedo-to-mature green stage embryo, compared with endosperm and aerial tissues [21–23]. However, the precise dynamics of DNA methylation occurring during embryogenesis has not been examined. During embryogenesis, embryo accumulates reserves for later germination, then they transit to the desiccation phase where dehydration occurs and the seed becomes dormant [24]. The dormant dry seed is biologically quiescent but competent to germinate. The genome-wide distribution, density, and sequence context of DNA methylation in the dry Arabidopsis seed has not been examined, but hypermethylation in the developing embryo must be reprogrammed to levels observed in aerial tissues. Moreover, the timing of initiation and mechanisms controlling these events is unknown, although recently observed hypomethylation during germination of rice seeds [25].

Here, we described the dynamics of global reprogramming of DNA methylation during seed development and germination in Arabidopsis. During seed development, extensive CHH methylation occurs within TEs in an RdDM-dependent and CMT2-dependent manner. During germination, hypermethylation in the dry seed is reprogrammed by passive CHH demethylation in a ROS1-independent manner. ROS1-dependent DNA demethylation is active in the late embryogenesis stage, where it antagonizes RdDM in the embryo and is responsible for establishment of endosperm specific DNA methylation. The dynamic global gain and subsequent loss of DNA methylation suggests a role of this epigenetic program in seed dormancy.

## Results

### Dynamic CHH methylation during embryogenesis and germination

To better understand the dynamics of DNA methylation variation through the plant life cycle, we compared single-base resolution methylomes of seeds at embryogenesis and germination stages in Arabidopsis (Additional file 1: Table S1). Germination methylomes were generated from Col-0 dry seeds and seedlings at 0–4 days after imbibition for 4 days (DAI) by MethylC-seq [26, 27]. These data were compared with publicly available methylomes of Ws-0 developing seeds from globular stage (4 days after pollination [DAP]), linear cotyledon stage (8 DAP), mature green stage (13 DAP), post-mature green stage (18 DAP), and dry seed (Ws-0), leaf [28], flower bud [26], microspore [17], sperm [19], vegetative nucleus [19], hand dissected embryo and endosperm (mid-torpedo to early-maturation stage; 7–9 DAP) [22], and columella root cap [29].

**Fig. 1:**
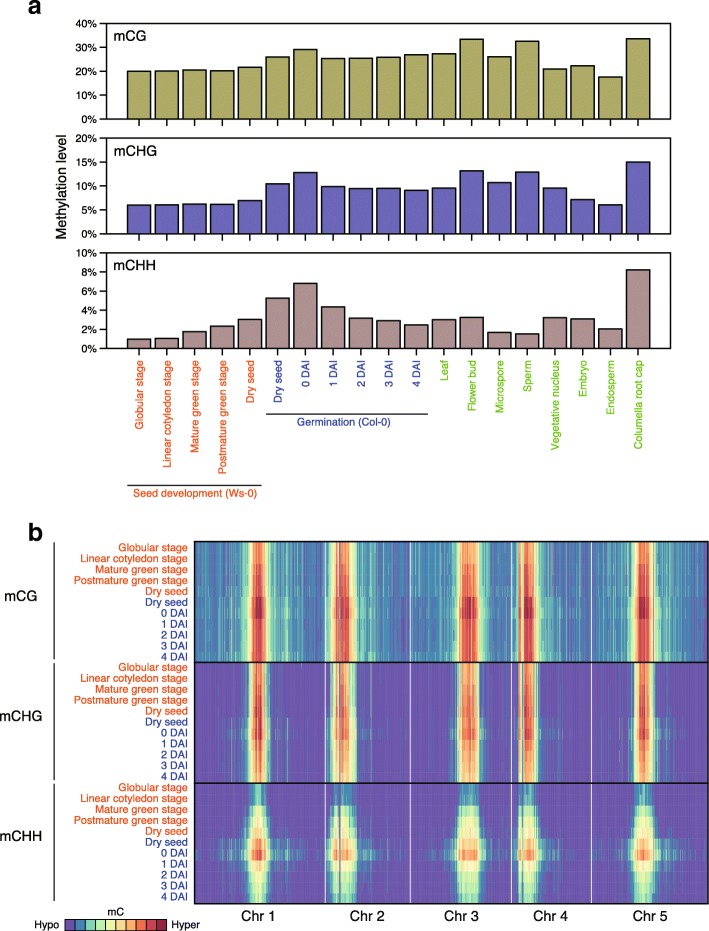
Genome-wide methylation dynamics during seed development and germination. **a** Genome-wide weighted methylation levels of developing seeds (Ws-0 background), germinating seeds (Col-0), leaf [28], flower bud [26], microspore [17], sperm [19], vegetative nucleus [19], hand dissected embryo and endosperm (mid-torpedo to early-maturation stage; 7–9 DAP) [22], and columella root cap [29] in each sequence context. (*Top*) mCG, (*middle*) mCHG, (*bottom*) mCHH. **b** *Heatmaps* showing methylation levels of developing seeds and germinating seeds for each sequence context. (*Top*) mCG, (*middle*) mCHG, (*bottom*) mCHH

Global methylation analysis revealed that mCG and mCHG were most stable throughout seed development (Fig. 1a). Dry seed global mCHH levels (~3%) were twofold higher than globular and linear cotyledon stage mCHH levels (~1%). These results are consistent with active MET1, CMT3, and RdDM pathways during embryogenesis [23]. Hypermethylation was observed in all sequence contexts from post-maturation to dry stages indicating that RdDM, rather than MET1 or CMT3, is still active during desiccation until dormancy, since cell division and DNA replication do not take place at these stages.

A striking feature observed for the Col-0 dry seed methylome was the extensive hyper mCHH (Fig. 1a, Additional file 2: Figure S1). In fact, mCHH levels in dry seeds were higher than mCHH levels in all other tissues and cells, except for columella root cap. mCG and mCHG levels in dry seeds were similar to those in leaf, but lower than those of flower bud, sperm, and columella root cap. Interestingly, we observed that mC levels in all contexts were higher in 0 DAI seeds that were imbibed and stratified for four days, than in dry seed, suggesting that RdDM is active during stratification even at 4 °C. mC levels in all contexts dropped at 1 DAI. A decrease in the mCHH level continued until 4 DAI where the level was even more reduced than found in rosette leaves. After 1 DAI, the mCG level increased while the mCHG level marginally decreased.

The distribution of mC along chromosomes was analyzed in 100 kb bins (Fig. 1b). mC was enriched in all sequence contexts at centromeres and peri-centromeres, although mCG was also broadly distributed in the chromosome arms. The gain and subsequent loss of mC during seed development and germination, respectively, occurred within these regions.

### Dynamic DNA methylation change occur within TEs

**Fig. 2:**
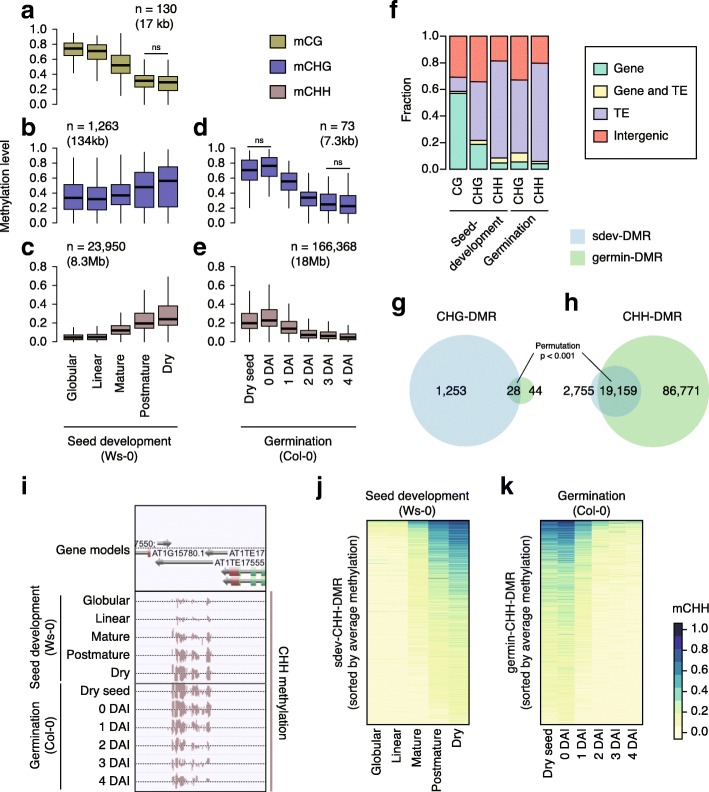
Dynamic epigenetic reconfiguration during seed development and germination. **a**–**c** Methylation levels within sdev DMRs. **d**, **e** Methylation levels germin DMRs. **a** mCG within CG DMR, **b**, **d** mCHG within CHG DMR, **c**, **e** mCHH within CHH DMR. Number of DMRs and total length of DMRs are indicated. Wilcoxon rank sum tests were applied to subsequent stages. Only non-significant pairs were indicated by “ns,” otherwise the methylation levels were significantly different (*p* < 0.05) between stages. **f** The fraction of genomic features overlapping with DMRs. **g**, **h** *Venn diagrams* showing overlap between sdev DMRs and germin DMRs. **g** CHG DMR and (**h**) CHH DMR. Overlap between sdev DMRs and germin DMRs were significant (permutation test with 1000 trials: *p* < 0.001 and *p* < 0.001, respectively). **i** A representative TE showing the gain of CHH methylation during seed development (*top*) and the loss of CHH methylation during germination (*bottom*). **j**, **k** *Heatmaps* showing CHH methylation levels within sdev DMRs and germin DMRs, respectively. DMRs were sorted by average methylation levels

To examine local DNA methylation changes, we identified seed-development-related (sdev) differentially methylated regions (DMRs) and germination-related (germin) DMRs by combining differentially methylated cytosine sites within 100 bp using the methylpy pipeline [30]. Sdev DMRs were called from comparison between Ws-0 methylomes of developing seed at globular stage, linear cotyledon stage, mature stage, post mature green stage, and dry seed. Germin DMRs were called from comparison between Col-0 methylomes of dry seed and germinating seed at 0-4 DAI. We found 25,343 sdev DMRs and 166,441 germin DMRs in total (Additional file 3: Table S2). Over 95% of DMRs were CHH DMRs, whereas no germin-CG DMRs were identified that met our criteria. Sdev-CHH DMRs and germin-CHH DMRs covered 8.3 Mb (7%) and 18 Mb (15%) of the reference genome, respectively (Fig. 2c and e). Whereas sdev-CG, sdev-CHG, and germin-CHG DMRs covered less than 0.1% of the reference genome (Fig. 2a, b, and d). Overall, mCG levels within sdev-CG DMRs decreased during seed development, but mCHG and mCHH levels within sdev-CHG and sdev-CHH DMRs increased as maturation proceeded (Fig. 2a–c). mCHH levels within germin-CHH DMRs were higher in 0 DAI seed than in dry seed (Additional file 4: Table S3; Wilcoxon rank sum test: *p* = 0), suggesting that these DMRs were further methylated during stratification (Fig. 2e). Then mCHG and mCHH levels within germin-CHG and germin-CHH DMRs during 0–3 DAI and during 0–4 DAI, respectively (Fig. 2d and e, Additional file 4: Table S3; Wilcoxon rank sum test: *p* < 0.05). We next examined genomic features overlapping with DMRs (Fig. 2f). We found that 60% of sdev-CG DMRs overlapped with protein-coding genes and 10% overlapped with TEs, whereas 19% of sdev-CHG DMRs overlapped with protein-coding genes and 44% with TEs. Finally, 73% of sdev-CHH DMRs overlapped with TEs while similar level, germin-CHG DMRs (60%) and germin-CHH DMRs (74%) overlapped with TEs, respectively.

Twenty-eight sdev-CHG DMRs and germin-CHG DMRs overlapped (permutation test: *p* < 0.001), whereas 82% (19,159) of sdev-CHH DMRs overlapped with germin-CHH DMRs (permutation test: *p* < 0.001) (Fig. 2g–i). Discrepancy in the number of sdev and germin DMRs is likely a consequence of the different accessions used to analyze seed development (Ws-0; from public database) and germination (Col-0; our study), due to following observations. First, Ws-0 seed development methylomes had no data (sequence reads) for 23,500 germin-CHH DMRs, even though Ws-0 methylomes (×24 ~ ×31 per strand) had higher coverage than Col-0 (×5 ~ ×9 per strand) methylomes, suggesting that these regions are absent from Ws-0 genome. Second, mCHH levels within sdev-specific and germin-specific CHH DMRs in Ws-0 dry seed and Col-0 dry seed differed more than those within sdev-common and germin-common CHH DMRs, suggesting these sdev-specific and germin-specific CHH DMRs are accession specific (Additional file 2: Figure S2). Nevertheless, we observed that mCHH levels within germin-specific CHH DMRs increased during seed development in Ws-0 and mCHH levels within sdev-specific CHH DMRs decreased during germination in Col-0 (Additional file 2: Figure S2). Again, virtually all sdev-CHH DMRs showed increasing mCHH levels toward maturation, whereas germin-CHH DMRs showed decreasing mCHH levels during germination (Fig. 2j and k). Collectively, the mCHH gained within TEs during seed development was lost during germination.

To examine whether DMRs affect the expression of nearby genes, we performed messenger RNA sequencing (mRNA-seq) analysis for dry seed and seeds/seedlings at 0, 1, and 2 DAI (Additional file 5: Table S4). As germination proceeded, more genes were expressed (FPKM > 1; Additional file 5: Table S4). Germination-expressed genes were classified into ten clusters based on their level of expression (Additional file 2: Figure S3A). Genes in clusters 5 and 9 were induced during the germination period. Twenty-seven percent (837/3144) and 25% (369/1485) of genes in clusters 5 and 9 were associated with germin-CHH DMRs, whereas 23% (4791/20,836) of all expressed genes were associated with germin-CHH DMRs (Additional file 2: Figure S3B and Additional file 6: Table S5). Therefore, germin-CHH DMRs were slightly enriched nearby germination-regulated genes in clusters 5 and 9 (Additional file 2: Figure S3B; fold enrichment: 1.2 and 1.1; one-sided Fisher’s exact test: *p* = 1.3e-07 and 0.043, respectively), compared with all expressed genes. This suggests that hypermethylation during seed development and hypomethylation during germination are at least partially associated with germination-related gene expression.

### RdDM and CMT2 pathways are active during seed development

**Fig. 3:**
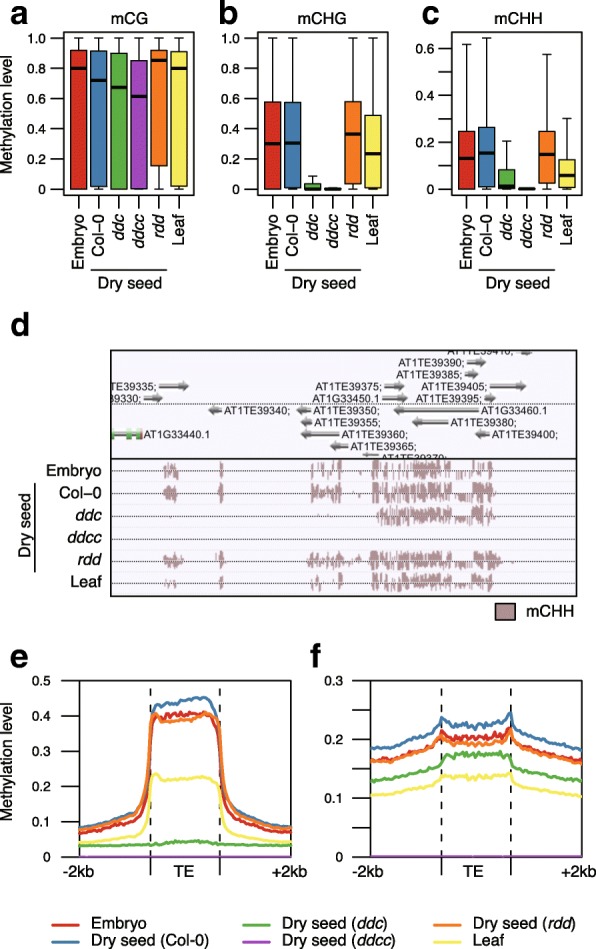
RdDM-dependent and CMT2-dependent hypermethylation of transposons occur during seed development. **a**–**c** *Boxplots* showing methylation levels within TEs in embryo (Col-0) at mid-torpedo to early-maturation stage, in dry seeds of WT (Col-0), *ddc*, *ddcc*, and *rdd*, and in leaf (Col-0): (**a**) mCG, (**b**) mCHG, (**c**) mCHH. **d** A browser snapshot of CHH methylation levels within TEs. Some TEs lost CHH methylation both in *ddc* and *ddcc*, and others did only in *ddcc*. **e**, **f** CHH methylation patterns across RdDM-targeted TE and CMT2-targeted TE, respectively. Embryo and leaf methylome data are obtained from [22] and [48], respectively

To elucidate the pathway responsible for TE hypermethylation during seed development, we compared dry seed methylomes from wild-type (WT) (Col-0), *drm1 drm2 cmt3* (*ddc*) triple mutants [31], and *drm1 drm2 cmt2 cmt3* (*ddcc*) quadruple mutants [8] (Fig. 3a–c). *MET1* transcripts, *CMT3* transcripts, *DRM2* transcripts, and their products are abundant in developing embryos, whereas only a marginal level of *CMT2* expression was observed [23]. Therefore, only RdDM is thought to be responsible for mCHH hypermethylation during embryogenesis. Whereas mCG levels within TEs mildly decreased in *ddc* and *ddcc* mutants (Wilcoxon rank sum test: *p* = 2.6e-38 and 2.5e-180, respectively), mCHG and mCHH levels drastically decreased, compared with Col-0 (Wilcoxon rank sum test: *p* = 0 for all comparisons). Interestingly, *ddcc* had lower mC levels within TEs in all contexts compared with *ddc* (Additional file 2: Figure S4; Wilcoxon rank sum test: *p* = 1.7e-38, 8.0e-205 and 0 for mCG, mCHG and mCHH, respectively). Indeed, we observed TEs substantially retain high mCHH levels in *ddc* triple mutants that are lost in *ddcc* quadruple mutants (Fig. 3d), suggesting CMT2 activity during seed development, in contrast to the previous report [23].

Next, we compared fluctuation in mCHH levels across the bodies of TEs in dry seeds of WT and mutant plants. To clarify the contribution of each pathway to TE methylation during seed development, we considered RdDM-targeted TEs and CMT2-targeted TEs (Fig. 3e and f). RdDM-targeted TEs and CMT2-targeted TEs were designated as TEs affected in *drm1 drm2* and in *cmt2* in leaf, respectively [32]. Although the overall methylation patterns along TE bodies in the embryo at mid-torpedo to early-maturation stage and dry seed were similar, hypermethylation of TEs was clearly evident in dry seed methylomes. The edges of CMT2-targeted TEs have sharp peaks of mCHH due to RdDM [7]. These peaks were pronounced in both embryo and dry seed, compared with leaf, indicating enhanced RdDM activity in these tissues (Fig. 3f). mCHH levels within RdDM-targeted TE bodies dropped to the same levels outside TE bodies and it was completely lost in *ddc* and *ddcc* (Fig. 3e). mCHH levels within CMT2-targeted TE bodies decreased in *ddc*, but substantial mCHH remained (Fig. 3f). mCHH peaks at the edge of CMT2-targeted TEs disappeared in *ddc* dry seeds. In contrast, *ddcc* dry seeds lose mCHH within CMT2-targeted TEs. Therefore, our data clearly show that CMT2 as well as RdDM is required for DNA methylation during seed development.

**Fig. 4:**
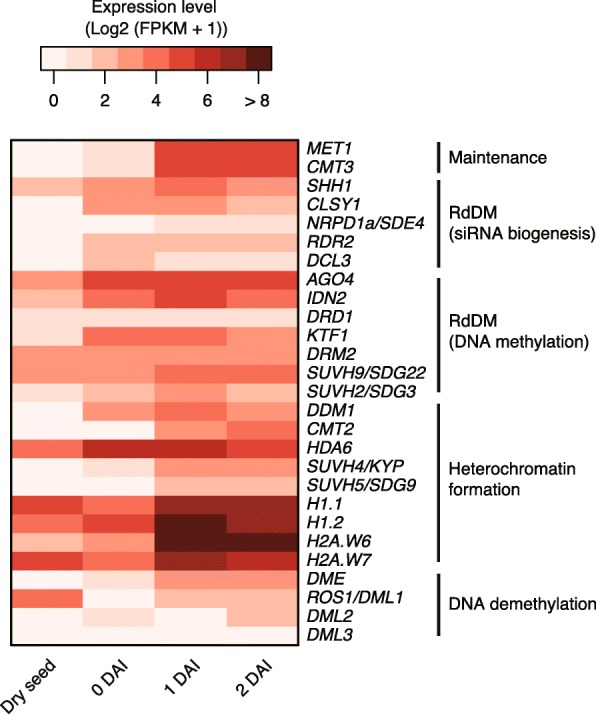
Expression levels of genes involved in DNA methylation/demethylation and silencing pathway components in germinating seeds. A *heatmap* of gene expression levels of DNA methylation related components in each pathway showing distinct gene expression trends for each module. Expression levels were shown as log2 (FPKM + 1)

Dry seeds store substantial levels of RNA transcripts for components of DNA methylation in the RdDM pathway, including *DRM2* (Fig. 4). In contrast, almost no transcripts for components of DNA methylation maintenance, small interfering RNA (siRNA) biogenesis or heterochromatin formation were detected in dry seed, although these genes are expressed during seed development, at least until mature green embryo stage (Fig. 4 and Additional file 2: Figure S5). This suggests that *MET1*, *CMT3*, *CMT2* pathways, and siRNA biogenesis pathway are active only before desiccation, but *DRM2* is active throughout seed development including the desiccation stage.

### Global demethylation during germination does not depend on DNA demethylases

**Fig. 5:**
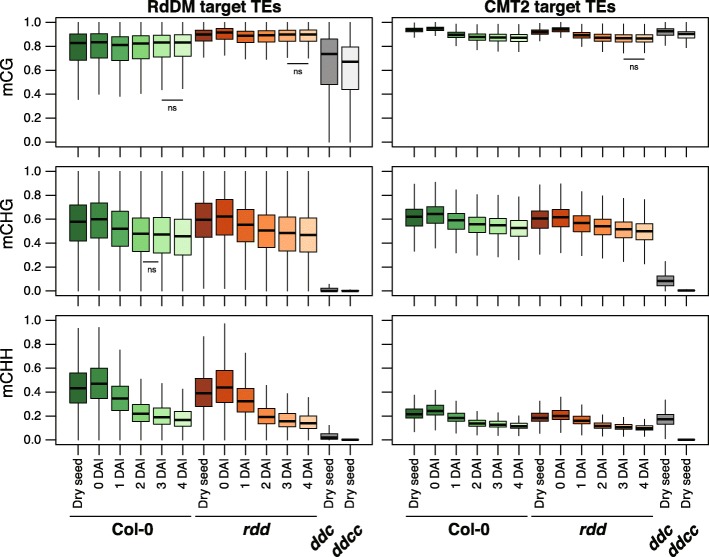
Passive demethylation during germination. *Boxplots* showing methylation levels within RdDM-targeted and CMT2-targeted TEs during germination. CHH methylation levels decrease during germination both in Col-0 and *rdd. DAI* days after 4 days imbibition at 4 °C in the dark. Wilcoxon rank sum tests were applied to subsequent stages. Only non-significant pairs were indicated by “ns,” otherwise the methylation levels were significantly different (*p* < 0.05) between stages. Methylation levels between Col-0 and *rdd* at all time points were significantly different (Wilcoxon rank sum test: *p* < 0.05; not indicated)

DME, a DNA demethylase, is responsible for local DNA demethylation in the pollen vegetative nucleus and endosperm central cells [19]. These demethylation events occur in companion cells and are involved in genomic imprinting and transposon silencing in the neighboring gamete cells [10, 19, 33]. To examine the possible involvement of DNA demethylases in global demethylation during germination, we compared the methylation levels within TEs of germinating seeds/seedlings of WT (Col-0) and *ros1 dml2 dml3* (*rdd*) triple demethylase mutant plants [12] (Additional file 2: Figure S5). At all time points, mCG and mCHG levels within RdDM-targeted TEs were slightly higher in *rdd* than in WT, whereas mCHH levels within RdDM-target TEs and mCG, mCHG and mCHH levels within CMT2-targeted TEs were slightly higher in WT than in *rdd* (Fig. 5, Additional file 4: Table S3; Wilcoxon rank sum test *p* = 2.9e-03 ~ 6.7e-278). Overall, Col-0 and *rdd* showed similar methylation level changes (Fig. 5). Germinating seed (0 DAI and 1 DAI) methylation levels, in all sequence contexts, were slightly higher and lower than found in dry seed, respectively. mCG levels within RdDM-targeted TEs were slightly re-elevated to the similar levels in dry seed between 2 and 4 DAI. In contrast, mCG levels within CMT2-targeted TEs marginally but further decreased between 2 and 4 DAI. mCHG and mCHH levels within both RdDM-targeted TEs and CMT2-targeted TEs decreased during germination. Remarkably, more than half of all mCHH sites within both RdDM-targeted TEs and CMT2-targeted TEs were lost in the period from germination until 4 DAI. These results indicate that ROS1, DML2, or DML3 are not involved in global demethylation during germination. Indeed, *ROS1* and *DML2* are very weakly expressed while *DML3* is not express during germination (Fig. 4). Rather, this global demethylation likely occurs in a passive manner by methylation dilution promoted by cell division, as suggested by the enrichment of cell division related genes in germination-related genes (clusters 5 and 9 in Additional file 2: Figure S3 and Additional file 7: Table S6). Relatively stable mCG and mCHG levels and dynamic reduction of mCHH levels suggest that CG maintenance by MET1 and CHG maintenance by CMT3 are active, whereas RdDM and CMT2 pathways for mCHH establishment and maintenance are not fully active during germination.

**Fig. 6:**
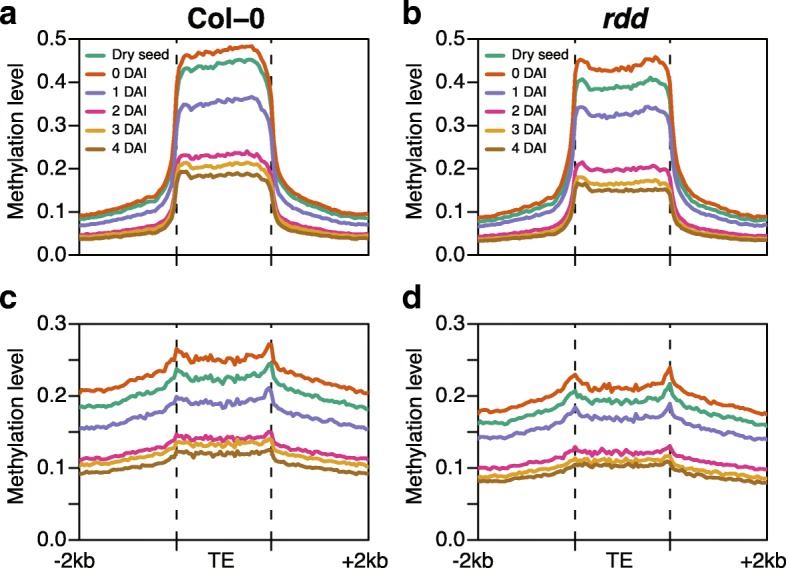
Changing transposon CHH methylation profiles in germinating seeds. **a**, **b** Averaged CHH methylation patterns across RdDM-targeted TEs. **c**, **d**. Averaged CHH methylation patterns across CMT2-targeted TEs. **a**, **c** Col-0 germinating seeds. **d**, **e** *rdd* germinating seeds. *DAI* days after 4 days imbibition at 4 °C in the dark

Next, we examined the mCHH pattern changes across TEs during germination (Fig. 6). Col-0 and *rdd* dry seeds showed slightly different mCHH patterns across RdDM-targeted TEs (Fig. 3e). Compared with WT, mCHH levels dropped near the center of RdDM-targeted TE bodies in *rdd* mutants. However, similar mCHH patterns were observed within RdDM-targeted TEs in 4 DAI WT (Col-0) and *rdd* plants, suggesting that reconfiguration could reset aberrant mCHH patterns caused by loss of DNA demethylases (Fig. 6a and b). Although the distribution of mCHH within CMT2-targeted TEs was similar in WT and *rdd* dry seeds, Col-0 TEs showed higher mCHH levels (Fig. 3f). Both Col-0 and *rdd* had mCHH peaks at the edges of CMT2-targeted TEs. However, peaks at the edges of CMT2-targeted TEs, a consequence of RdDM (Fig. 3f), become less pronounced at 3 DAI in both in Col-0 and *rdd* (Fig. 6c and d), indicating that the rate of mC loss was slower inside TE bodies than at the edges of TE bodies. Since global demethylation is likely passive, this suggests that CMT2 activity started to recover at this stage, whereas RdDM must still be inactive. Indeed, *CMT2* expression initiated at 1 DAI, but siRNA biogenesis components expression stayed low even at 2 DAI, whereas *DRM2* was expressed at a steady level (Fig. 4).

Collectively, our data suggest a global passive demethylation reprograms CHH hypermethylation in the dry seed during the four days post-germination period.

### ROS1 is active in developing seed during late embryogenesis

**Fig. 7:**
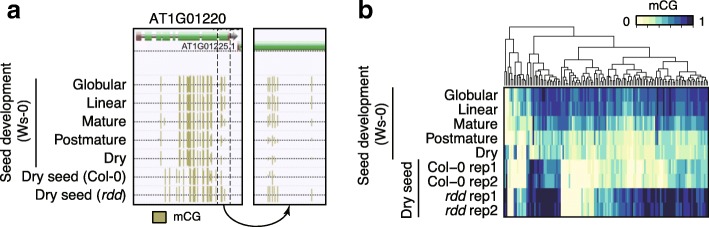
ROS1-dependent seed development related CG DMRs. **a** A representative snapshot of sdev-CG-DMRs during seed development (Ws-0) and dry seeds of WT (Col-0) and *rdd*. (*Right*) An enlarged view of the area indicated by the *dotted line* in the *left panel*. Heights of yellow ticks show the methylation level for each CG site. **b** A *heatmap* showing mCG levels within sdev-CG DMRs

Overall, active methylation occurs during embryogenesis and passive demethylation occurs during germination. However, mCG levels within sdev-CG DMRs decreased during seed development, especially between mature and post-mature stages (Fig. 2a; Wilcoxon rank sum test: *p* = 1.7E-19). Nearly 60% of CG DMRs overlapped with genes. mCG in gene bodies, so-called gene body methylation (gbM), is stable because mCG is maintained by MET1 DNA methylase during DNA replication. Since cell division does not occur in the mature stage embryo, we hypothesized that mCG hypomethylation within sdev-CG DMRs was caused by active demethylation. RNA sequencing (RNA-seq) revealed the presence of *ROS1* transcripts, but low or absent expression of *DME*, *DML2*, *DML3* transcripts in dry seeds, suggesting that ROS1 is active during late embryogenesis (Fig. 4). We compared mCG levels in dry seed of Col-0 and *rdd* within sdev-CG DMRs. CG hypomethylation within sdev-CG DMRs was retained in dry seed of Col-0, but not in *rdd*. mCG levels in dry seed of *rdd* were higher than in dry seed of Col-0 (*rdd* - Col-0 > 0.2) in 75% (97/130) of sdev-CG DMRs (in both replicates) (Fig. 7a and b). It is unclear whether ROS1 is active throughout seed development, but our data showed ROS1 expression and activity in developing seed, at least in the late stage of embryogenesis, generates sdev-CG DMRs.

### *rdd* seeds show increased methylation at endosperm-specific hyper-DMRs

**Fig. 8:**
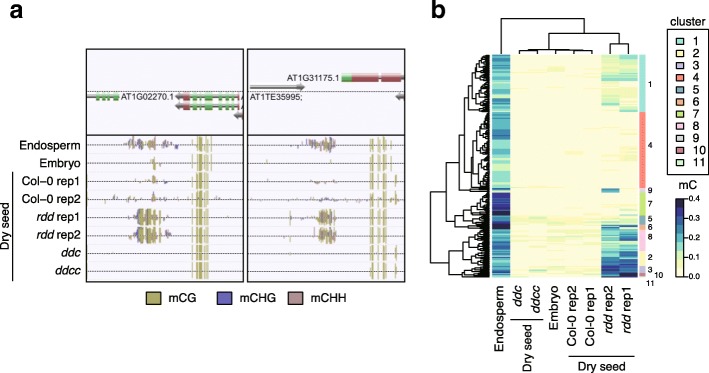
A half of endo-DMRs are methylated in *rdd* dry seed. **a** Representative snapshots of endo-DMRs. **b** A *heatmap* showing methylation levels within endo-DMRs. Hierarchical clustering classified endo-DMRs into 11 clusters. Numerals at the *right* side of the heatmap indicate cluster number where each endo-DMR belongs to. Endo-DMRs in clusters 1, 2, 3, 6, 8, 10, and 11 are methylated in *rdd* dry seed but not in WT dry seed. Embryo and endosperm methylome data are obtained from Hsieh et al.

DME and ROS1 are closely related DNA demethylases, but they are active at distinct sites, even in developing seeds. DME locally demethylates TEs in the endosperm and demethylated TEs are transcribed, leading to siRNA production [19]. These siRNAs are hypothesized to be transported to the embryo and reinforce TE methylation in the embryo. We compared methylomes in dry seed of Col-0, *rdd*, *ddc*, and *ddcc* and in embryo and endosperm at mid-torpedo to early-maturation stage of Col-0. We identified 44,554 DMRs in all contexts (C-DMRs) among these methylomes (Additional file 4: Table S3). Among these, we found 194 endosperm-specific hyper-DMRs (endo-DMRs) that were methylated in the endosperm but not in the embryo or in dry seeds of Col-0 (Fig. 8). Hierarchical clustering based on differences in DNA methylation levels classified endo-DMRs into 11 clusters (Fig. 8b). Methylation levels within endo-DMRs in clusters 1, 2, 3, 6, 8, 10, and 11 were increased in dry seed of *rdd*, compared with in dry seed of Col-0, suggesting that ROS1 is required to demethylate these regions during seed development.

## Discussion

DNA methylation profiles showed substantial variation between tissues, somatic cell types, and reproductive cell types [17, 19, 21, 22, 26, 29, 34–36]. DNA methylation is reprogrammed in the pollen and the central cell. However, the dynamic features of DNA methylation reprogramming have not been measured during seed development and germination. We describe developmental reprogramming of DNA methylomes during seed development and germination. The most striking feature of seed methylomes is the genome-wide dynamic gain and loss of mCHH during seed development and germination, respectively. Although we observed association between germin-CHH DMRs and germination-induced gene expression, hypermethylation in dry seed may be related to the halt of transcription in dry seed. One possible explanation is that genome-wide hypermethylation reinforces the packing of chromatin to prevent unfavorable induction of gene expression or TE activation. When conditions become favorable for germination, global DNA hypomethylation may release tightly packed chromatin and the expression of germination-related genes promoted. This idea is supported by the fact the heterochromatic chromocenters just after germination are smaller than those at three weeks after germination and during germination [37, 38]. It is also possible that the accumulation of mC would block the damage to genomic DNA which may causes mutation during seed dormancy, a period that can extend from days to centuries.

Genome-wide CHH hypermethylation was also observed in root columella cells [29]. In the columella cells, loosened heterochromatin allows access of RdDM components to the heterochromatic regions, leading to excess 24 nt small RNAs and hypermethylation. In contrast to columella cells, siRNA biogenesis is less active during late seed development, because components of siRNA biogenesis are not expressed (Fig. 4). Therefore, 24-nt siRNAs produced in seed during the early to mid embryogenesis stages may be stored during the late embryogenesis stages. This may be supported by the observation that the fraction of 24-nt small RNA is larger than other sizes of small RNA in maize dry seed [39]. Upon complete desiccation, DNA methylation may be halted. During stratification at low temperature after imbibition, stored siRNAs would keep recruiting DRM2 to target loci under unfavorable condition for other biological processes, leading to hypermethylation at 0 DAI. This buffering mechanism would be of benefit when dry seeds are exposed to transient germination conditions to recover to a pre-germinating state. It is also possible that RdDM acts at CMT2 targeted TEs during late seed development, as in columella cells, possibly because transcript levels of heterochromatin formation related components are much lower in dry seed, compared with imbibed seed (Fig. 4). In support of this model, CMT2 targeted TEs are also hypermethylated at 0 DAI, when only a trace of *CMT2* transcript is observed. One enigma is that RdDM and CMT2 suddenly lose their activity upon germination, although RdDM components and CMT2 are expressed during germination, whereas MET1 and CMT3 are active during early germination. Further experiments will be needed to elucidate what antagonizes RdDM and CMT2 activities during early phases of germination. One possibility is that the rapid rate of cell division rate exceeds the ability to de novo re-methylation.

Endosperm-specific methylation patterns have been identified [22], but the mechanism of how these patterns are established is still unknown. We found that ROS1 is required for demethylation of endosperm-specific methylated regions in the seed. This raises the possibility of an association between ROS1 activity and endo-DMRs. Future studies with embryo-specific and endosperm-specific methylome analysis using *rdd* mutants are needed to further explore this hypothesis.

## Conclusion

Our study has revealed dynamic genome-wide reconfiguration of DNA methylation during seed development and seed germination in Arabidopsis. During seed development, an extensive gain of mCHH was observed, especially within TEs. This active hypermethylation continued during stratification, but was immediately reset upon germination. Hypermethylation during seed development relied on both RdDM and CMT2 pathways, whereas hypomethylation during germination occurred via a passive mode (an absence of methylation maintenance). Dormancy, the bridge between seed development and germination, is an important agronomic trait for many crops, closely associated with pre-harvesting sprout and synchronized germination. The data presented in this study can be served as a resource to address further studies of the role of DNA methylation in the association with dormancy. All methylome data analyzed in this study can be visualized using the Arabidopsis seed methylomes browser (http://neomorph.salk.edu/Arabidopsis_seed_methylomes.php).

## Methods

### Plant materials and growth condition

Col-0, *ddc* triple mutant, and *rdd* triple mutant dry seeds were used for methylome analyses. Col-0 and *rdd* dry seeds were sterilized with bleach and plated on half strength Linsmaier and Skoog medium supplemented with 1% agar. After four days stratification in the dark at 4 °C, plants were grown under long day condition at 22 °C.

### MethylC-seq

DNA was extracted from dry and germinating seeds using modified CTAB method [40]. One microgram genomic DNA was used for library preparation as described previously [26, 27]. Samples were sequenced with an Illumina HiSeq2500 instrument. Raw methylome data for Ws-0 seed development and *ddcc* mutant dry seed were obtained from the Gene Expression Omnibus (accession numbers: GSE68132 and GSE68131). Read mapping and base calling were performed as described previously [41], except that the reads were mapped against the C-to-T converted TAIR10 reference genome. The bisulfite non-conversion rate was calculated by the total number of cytosine base calls divided by the total coverage at cytosine positions in the naturally unmethylated chloroplast genome.

### Identification of differentially methylated regions

DMRs were identified using the methylpy pipeline [30] (https://bitbucket.org/schultzmattd/methylpy). In brief, differentially methylated sites (DMSs) were identified by root mean square tests with false discovery rate at 0.01, using 1000 permutations. Cytosine sites at least with 5 reads were examined for differential methylation. Then, DMSs within 100 bp were collapsed into DMRs. DMRs for mCG (CG DMRs), CHG DMRs, and CHH DMRs with fewer than eight, four, and four DMSs, respectively, were discarded in following analysis. In addition, CG DMRs, CHG DMRs, and CHH DMR candidate regions with less than 0.4, 0.2, and 0.1 differences between maximum and minimum methylation levels, respectively, were discarded. We compared seed development methylomes and germination methylomes separately, because global methylation levels are much higher in Col-0 than in Ws-0 [42]. Methylation levels were calculated as weighted methylation levels: the frequency of C base calls at C sites within the region divided by the frequency of C and T base calls at C sites within the region [43].

### Sdev - DMRs

Methylomes of Ws-0 seeds with globular (4 DAP), linear cotyledon (8 DAP), mature green (13 DAP), post mature green (18 DAP), and dry stage were used for differential methylation analysis. For heatmap analysis, R function heatmap.2 was used.

### Germin - DMRs

Two replicates methylomes of Col-0 dry seed and germinating seeds after days 0, 1, 2, 3, and 4 after four-day stratification were used for differential methylation analysis. Boxplots showed average methylation levels of two replicates, because methylation levels within TEs were highly concordant between two replicates.

### Endo-DMRs

Methylomes of embryo and endosperm at mid-torpedo to early-maturation stage, dry seed from Col-0, and dry seed from *rdd*, *ddc*, and *ddcc* were subject to differential methylation analysis using all contexts of cytosines. DMRs where methylation level of endosperm - methylation level of embryo > 0.1, methylation level of embryo < 0.1 and methylation level of Col-0 dry seed < 0.1 were designated as endo-DMRs. Endo-DMRs were clustered and visualized with R function heatmap.2 included in gplots package.

To count overlapping sdev DMRs and germin DMRs, we merged overlapping sdev DMRs and germin DMRs and classified them into only sdev DMRs, both sdev and germin DMRs, and only germin DMRs. Permutation tests were applied to examine whether overlap between sdev DMRs and germin DMRs was significant. For each permutation, we shuffled the coordinates of sdev DMRs and germin DMRs. To calculate *p* value, the number of permutations when the overlapping frequency between shuffled coordinates exceeded the actual overlapping frequency was divided by the total number of permutations (1000 trials).

### Methylation analysis of genes and TEs

TAIR10 annotation for protein-coding genes and TEs were used for methylation analysis. For metaplots, TE body, 2 kb upstream, and 2 kb downstream regions were split into equally sized 40 bins each. The average weighted methylation level for each bin from all TEs was plotted. Only data for replicate 1 were shown.

### RNA-seq and microarray

RNA was extracted from dry seed and germinating seed using modified phenol-SDS methods [44]. A total of 200 ng of total RNA was used for library preparation using TruSeq stranded mRNA LT library preparation kit (Illumina) following the manufacturer’s instruction. Reads were mapped to TAIR10 reference genome using TopHat2 (v2.0.8) with parameters (--library-type = fr-firststrand) [45]. Expression levels for each gene were calculated as FPKM (fragments per kilobase of exon per million fragments mapped) using cufflinks (v2.0.2) with TAIR10 annotation [46]. Expressed genes were designated as genes whose FPKM was > 1 at least in one sample. Only expressed genes were used in following analysis. Expressed genes were classified into ten clusters based on log2 (FPKM + 1) using R function kmeans (parameter: centers = 10, iter.max = 20). Gene Ontology analysis was performed using DAVID tools (https://david.ncifcrf.gov). Normalized microarray data generated by ATH1 GeneChip platform for developing seeds were obtained from Belmonte et al. [47].

### Associating DMRs with proximal genes

We designated that a gene and a DMR are associated if the DMR is located within 2 kb of gene upstream regions, gene bodies and 2 kb of gene downstream regions. Only the closest DMRs and genes were associated. When the distances between a DMR and flanking genes were equal, both gene pairs were kept.

## Additional files

Additional file 1: Table S1. Mapping metrics for MethylC-seq and RNA-seq. (XLSX 41 kb)

Additional file 2: Figures S1–5. This file contains Supplementary Figures S1-5 and Supplementary Figure legends. (PDF 761 kb)

Additional file 3: Table S2. Coordinates of DMRs and methylation levels within DMRs. (XLSX 20445 kb)

Additional file 4: Table S3. Lists of Wilcoxon rank sum test *p* values. (XLSX 40 kb)

Additional file 5: Table S4. Gene expression levels for expressed genes. (XLSX 1180 kb)

Additional file 6: Table S5. Genes associated with germin DMRs. (XLSX 4448 kb)

Additional file 7: Table S6. Gene ontologies enriched in transcriptome clusters. (XLSX 230 kb)

## Abbreviations

CMT2: CHROMOMETHYLASE 2
CMT3: CHROMOMETHYLASE 3
DAI: Days after four-day imbibition
DME: DEMETER
DML2: DEMETER LIKE 2
DML3: DEMETER LIKE 3
DMR: Differentially methylated region
DRM2: DOMAINS REARRANGED METHYLTRANSFERASE 2
FPKM: Fragments per kilobase of exon per million fragments mapped
mCG: CG methylation
mCHG: CHG methylation
mCHH: CHH methylation
MET1: METHYLTRANSFERASE 1
RdDM: RNA-directed DNA methylation
ROS1: REPRESSOR OF SILENCING 1
TE: Transposable element
WGBS: Whole genome bisulfite sequencing
