# Supplementary material for: Dynamic DNA methylation reconfiguration during seed development and germination
Source: Genome Biol. 2017 Sep 15;18:171. doi: 10.1186/s13059-017-1251-x (PMC5599895; doi:10.1186/s13059-017-1251-x)
Supplement: Supplementary file 2 — This file contains Supplementary Figures S1-5 and Supplementary Figure legends. (PDF 761 kb) [file 13059_2017_1251_MOESM2_ESM.pdf]

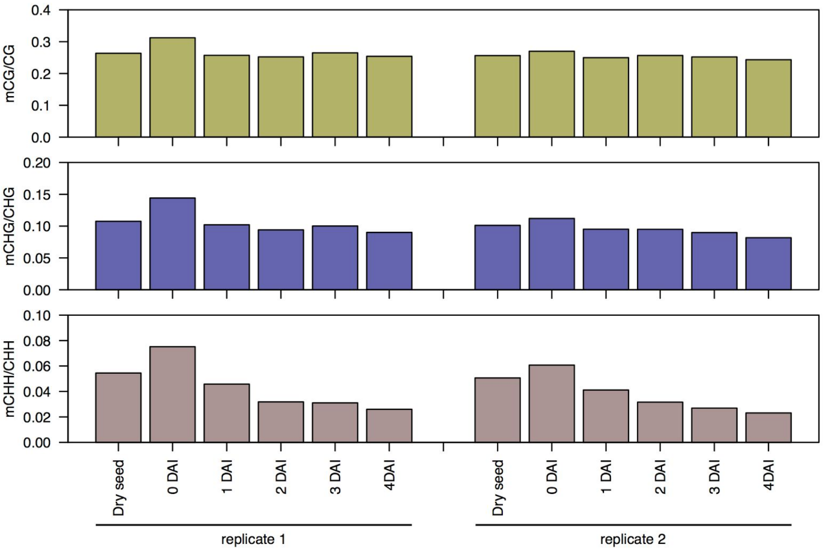

Supplemental Figure 1. Genome-wide methylation levels for each replicate of Col-0.

Genome-wide weighted methylation levels in germinating seed in Col-0 for two biological replicates. The top panel: mCG, the middle panel; mCHG, the bottom panel: mCHH.

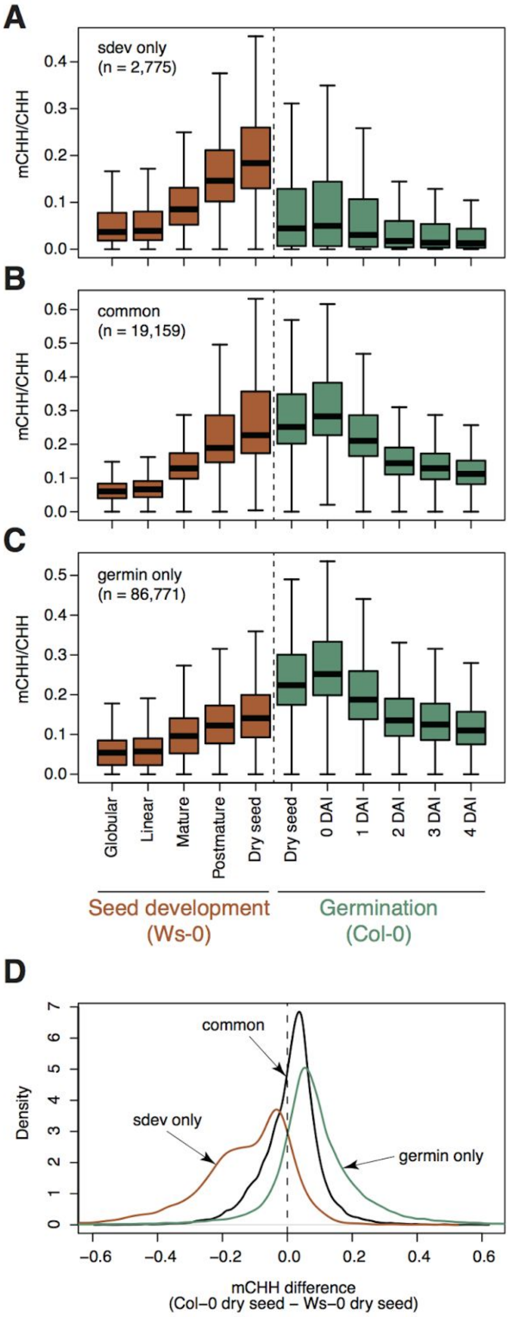

Supplemental Figure 2. Accession dependency of seed-development-related DMRs and germination-related-DMRs.

A. CHH methylation levels within sdev-CHH-DMRs which do not overlap with germin-CHH-DMRs (sdev only). **B.** CHH methylation levels within sdev- and germin- common CHH-DMRs (common). **C.** CHH methylation levels within germin-CHH-DMRs which do not overlap with sdev-CHH-DMRs (germin only). Numbers of DMRs were indicated. **D.** A density plot showing the distribution of CHH methylation differences between Col-0 dry seed and Ws-0 dry seed within each type of DMRs indicated in **A-C**.

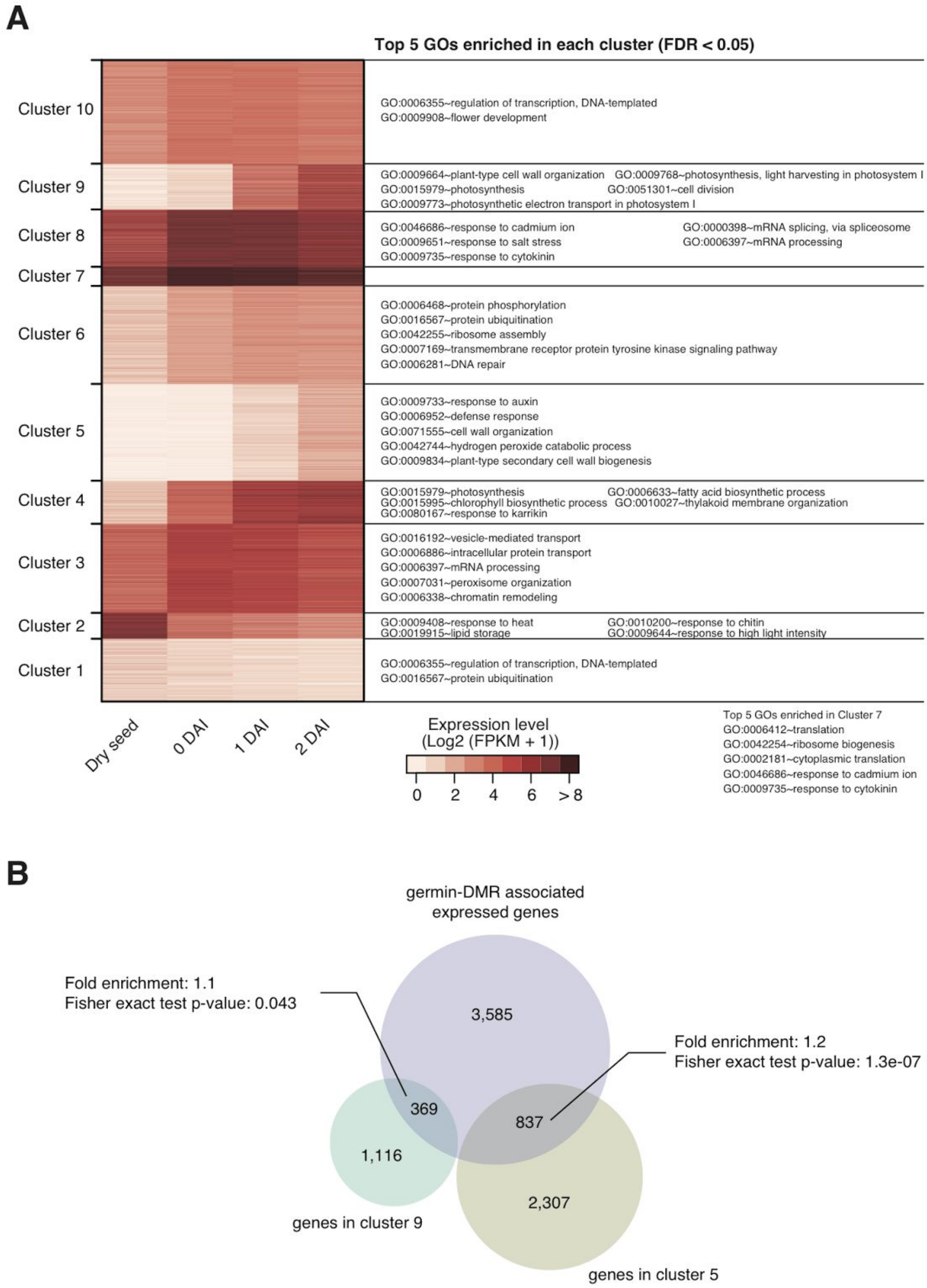

Supplemental Figure 3. Transcriptome changes during germination.

A. A heatmap showing the dynamics of transcriptomes during germination in Col-0. Expressed genes were classified into 10 clusters by kmeans, and top 5 Gene Ontologies enriched in each cluster are shown at the right side of the heatmap. **B.** Overlap between germin-CHH-DMRs and germination induced genes (clusters 5 and 6). Fold enrichments and Fisher exact test p-values are shown.

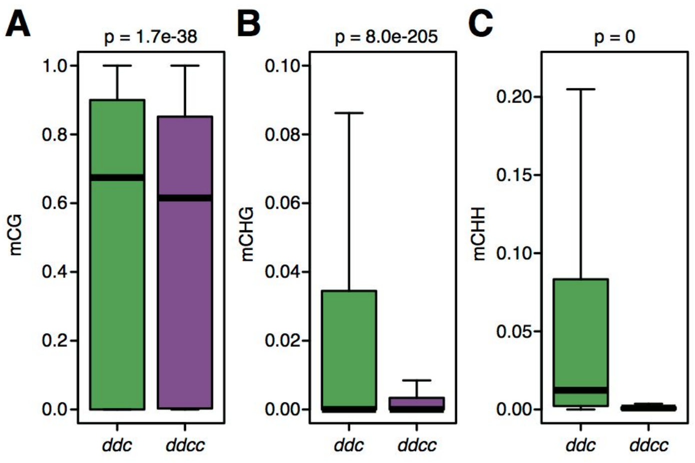

Supplemental Figure 4. Methylation levels within transposable elements in *de novo* DNA methylation pathway mutant dry seed. (Modified from Fig. 3A-C)

A - C. Boxplots showing methylation levels within TEs in dry seeds of *ddc* and *ddcc*. **A**: mCG, **B**: mCHG, **C**: mCHH. Wilcoxon rank sum test p-values are shown.

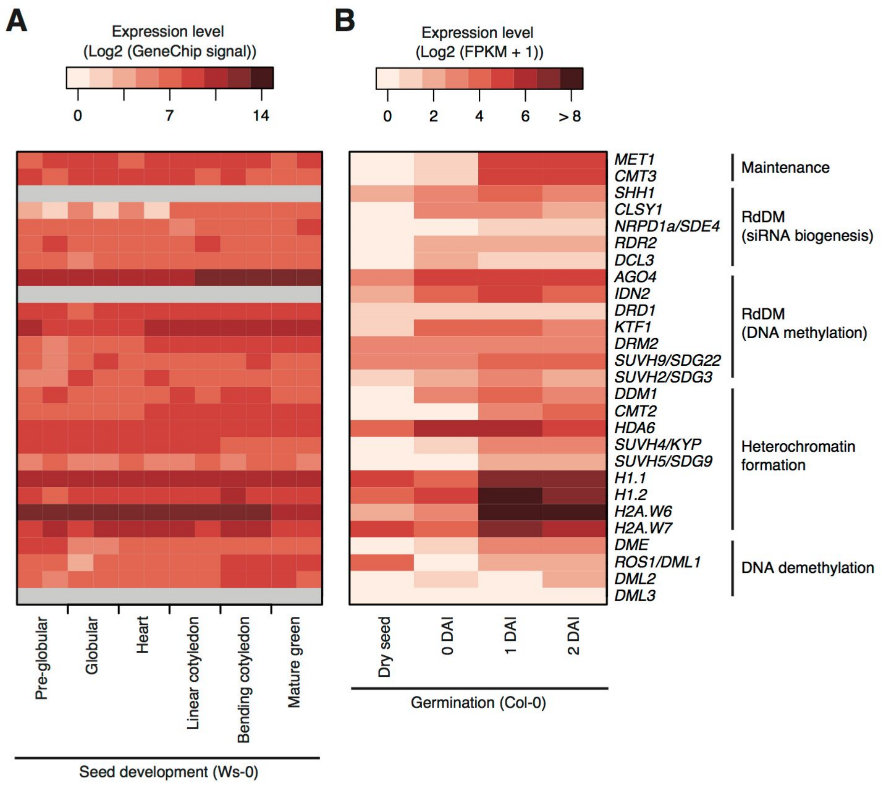

Supplemental Figure 5. Expression levels of genes involved in DNA methylation/demethylation and silencing pathway components in seeds.

A and **B.** Heatmaps of gene expression levels of DNA methylation related components in each pathway during seed development (**A**) and germination (**B**). B is the same as Fig 4 and shown for better comparison. Transcriptome analysis platforms are ATH1 GeneChip microarray (**A**) and RNA-seq (**B**). Expression levels were shown as log2 (GeneChip signal) for **A** and log2(FPKM + 1) for **B**. Data not available from microarray analysis are indicated by grey. Normalized microarray signal data are obtained from Belmonte et al., PNAS (2013).
